# Transcriptome and single-cell analysis reveal the contribution of immunosuppressive microenvironment for promoting glioblastoma progression

**DOI:** 10.3389/fimmu.2022.1051701

**Published:** 2023-01-05

**Authors:** Lulu Ni, Ping Sun, Sujuan Zhang, Bin Qian, Xu Chen, Mengrui Xiong, Bing Li

**Affiliations:** ^1^ Department of Basic Medicine, Jiangnan University, Wuxi, China; ^2^ Department of Pathology, The Affiliated Wuxi No. 2 People’s Hospital of Nanjing Medical University, Wuxi, China; ^3^ Institute of Science and Technology Information, Beijing Academy of Science and Technology, Beijing, China; ^4^ Department of Traditional Chinese Medicine, General Hospital of the Third Division of Xinjiang Production and Construction Corps, Tumushuke, China; ^5^ Department of Neurosurgery, The Affiliated Wuxi No. 2 People’s Hospital of Nanjing Medical University, Wuxi, China

**Keywords:** immunosuppression, GBM, WGCNA, TAM, single-cell, immunotherapy

## Abstract

**Background and objectives:**

GBM patients frequently exhibit severe local and systemic immunosuppression, limiting the possible efficacy of immunotherapy strategies. The mechanism through which immunosuppression is established in GBM tumors is the key to successful personalized immunotherapies.

**Methods:**

We divided GBM patients into subtypes according to the expression characteristics of the TME typing-related signature matrix. WGCNA analysis was used to get co-expressed gene modules. The expression activity of hub genes retrieved from co-expressed modules was validated in two single-cell datasets. Then, cell–cell interaction was calculated.

**Results:**

Four subtypes were identified in the TCGA and CGGA RNA-seq datasets simultaneously, one of which was an immunosuppressive subtype rich in immunosuppressive factors with low lymphocyte infiltration and an IDH1 mutation. Three co-expressed gene modules related to the immunosuppressive subtype were identified. These three modules are associated with the inflammatory response, angiogenesis, hypoxia, and carbon metabolism, respectively. The genes of the inflammatory response were mainly related to myeloid cells, especially TAM, angiogenesis was related to blood vessels; hypoxia and glucose metabolism were related to tumors, TAM, and blood vessels. Moreover, there was enhanced interaction between tumor cells and TAM.

**Discussion:**

This research successfully found the immunosuppressive subtype and the major cell types, signal pathways, and molecules involved in the formation of the immunosuppressive subtype and will provide new clues for the improvement of GBM personalized immunotherapy in the future.

## Graphical Abstract

Overview of the study design. **(A)** We firstly identified five GBM progression related pathways. By performing functional enrichment analysis on gene sets obtained from three perspectives, such as genes co-survival in TCGA-GBM and CGGA cohort, DEGs of high and low risk groups, and DEGs of IDH1 mutation compared with wild type, we identified five pathways significantly associated with poor prognosis in GBM patients. **(B)** Secondly, GBM TME-associated functional gene signatures were constructed. Based on the activity profile of these signatures, GBM patients were classified into four distinct subtypes and immunosuppressive subtypes were found. **(C)** the expression of hub genes from immunosuppressive subtypes were validated in three single-cell RNA-seq datasets, and cell types significantly associated with TME subtypes were identified. The interactions between certain cell types were also elaborated.

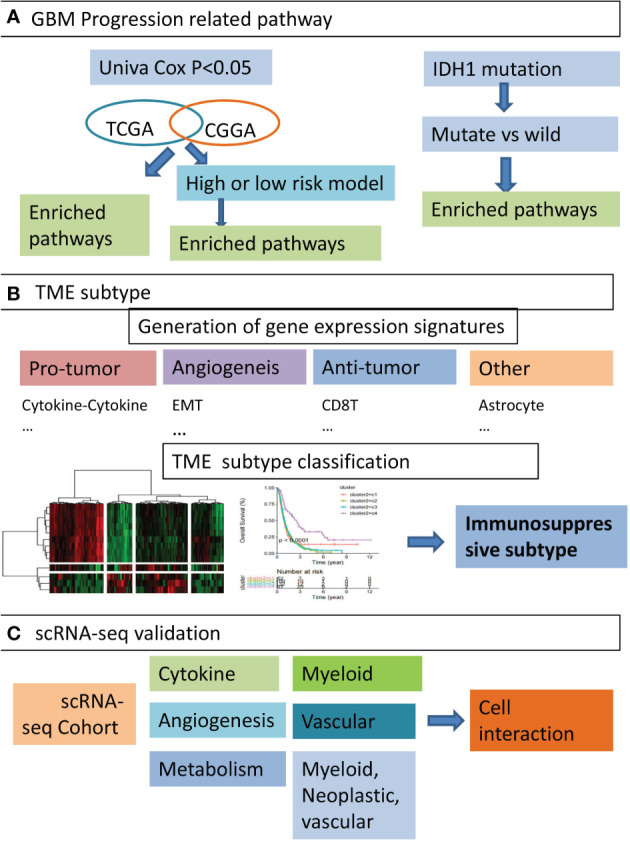

## Introduction

GBM is the most common primary tumor of the central nervous system (CNS) in adults and is notoriously difficult to treat because of its diffuse nature. The median survival time of GBM patients remains approximately 14–15 months after diagnosis (1, 2). Passage of systemically delivered pharmacological agents into the brain is largely blocked by the blood–brain barrier (BBB) (3). Although recent advances, including the addition of tumor-treating fields (TTF), have shown some modest benefits, the overall survival rate remains effectively unchanged (4). Effective new therapies are urgently required.

Immunotherapy has emerged as a promising treatment for some of the hardest-to-treat tumors, including metastatic melanoma. The general principle of immunotherapy is to fight immune suppression in the tumor microenvironment and activate the patient’s own immune system to kill the tumor. Successful cancer immunotherapy depends on the existence of an intact and functional immune system. However, GBM patients frequently exhibit severe local and systemic immunosuppression, which limits the possible efficacy of these therapeutic strategies (5). This apparent immunosuppression is a critical barrier to improving patient survival. Understanding the mechanism of establishing immunosuppression in GBM tumors is the key to successful personalized immunotherapy soon. However, the nature of these mechanisms remains surprisingly elusive.

The implications of specific immune cell types on GBM disease status were unknown. In most cancers, the presence of tumor-infiltrating lymphocytes (TILs) is positively correlated with the improvement of overall survival in patients, but the correlation between the presence of TILs and the improvement of overall survival in GBM patients has not been clearly established (6, 7). Myeloid cells, especially microglia and macrophages, in the tumor microenvironment regulate GBM progression and influence therapeutic outcomes (8). Besides, resident fibroblasts, endothelial cells, pericytes, and the extracellular matrix also contribute to cancer progression (9). Abnormal cytokine expression was found to be associated with glioma progression. Within the heterogeneous GBM microenvironment, tumor cells, normal brain cells, immune cells, and stem cells interact with each other through the complex cytokine network (10, 11). The formation of the GBM tumor microenvironment has been associated with specific mutations. For example, the IDH mutation has recently been found to be associated with decreased immune cell infiltration (12), whereas inactivated NF1 has been associated with increased macrophage infiltration (13). In addition, several major signaling pathways like NFκB, Wnt, and PI3K–AKT–mTOR are reported to be involved in the pathogenesis of GBM and have been used as therapeutic targets for GBM (14–16).

Based on the above knowledge, we constructed gene signatures that can be used to distinguish GBM samples, including tumor-promoting signaling pathways, angiogenesis-related genes, and various cell-characteristic gene signatures. GBM patients were classified into subtypes by clustering the expression characteristics of these gene signatures in each patient. Also, we found hub genes in each module through WGCNA analysis. Combined with published single-cell data, we identified cell types responsible for the abnormal expression of these hub genes and the pathways involved in this process. At the same time, the interactions between cell types and related ligand–receptor pairs were also studied. These analyses systematically analyzed the formation mechanism of the GBM microenvironment, especially the immunosuppressive microenvironment, and helped to find targets for immunotherapy.

## Methods

Publicly available GBM were obtained from The Cancer Genome Atlas (TCGA), and level 3 RNA-seq data for 167 GBM samples were downloaded from the UCSC Xena browser (https://xena.ucsc.edu/) (17). Corresponding clinical characteristics were obtained. Another 345 GBM samples with clinical information were provided by the Chinese Glioma Genome Atlas (CGGA). The detailed clinical and pathological characteristics of the TCGA-GBM and CGGA cohorts were summarized in **Supplementary Table 1**. Data on RNA-seq were transcripts-per-million (TPM) normalized and log2-transformed. Then, low expressed genes were eliminated.

Three GBM-related scRNA-seq datasets were retrieved from the GEO database (GSE117891 (n = 8), GSE84465 (n = 2), and GSE163120 (n = 12)) (18–20). After removing low-quality cells, followed by normalization and dimension reduction, Louvain clustering was used to group cells. GSE117891 and GSE84465 were integrated. Cell types were annotated using canonical marker genes. Additionally, malignant cells were defined by “InferCNV” (https://github.com/broadinstitute/InferCNV). All these were performed by Seurat (4.0) in the R package (21).

### Functional characterization of differential expression analysis (DEGs)

For the RNA-seq data, the DEseq2 R package was used. Genes with an FDR <0.05 and absolute fold change ≥1.5 were considered as differential expressed.

### Functional enrichment analysis

Functional annotation of DEGs was performed on the Kyoto Encyclopedia of Genes and Genomes (KEGG) and Gene Ontology (GO) classification databases. Enrichment analysis of GO categories was performed by the R clusterProfiler (v3.14.3) package, and pathway enrichment analysis was tested upon hypergeometric distribution by the R “phyper” function. GO categories with a false detection rate (FDR) of <0.05 were significantly enriched. The pathway with P <0.05 was enriched. Only those go categories or pathways containing ≥5 DEGs were retained.

### Weighted gene co-expression network analysis (WGCNA)

WGCNA was performed by the R package WGCNA (V1.69) (22). We use the log2-transformed TPM value as the normalized expression and filter out abnormal samples. According to the principle of scale-free network, coefficient β was set as 14. The parameter of network type was used with “signed” and “bicor” (double weighted correlation) to calculate the correlation adjacency matrix. Co-expression gene modules were identified by using dynamic tree cutting with the following major parameters: The main parameters minModuleSize and deepSplit were 30 and 1, respectively. The highly similar modules with the height of the module eigengene in the clustering lower than 0.2 were merged. A univariate Cox proportional hazard regression was performed on each gene module. Genes in each module with a p-value <0.05 were kept as modules’ survival-related genes. Those genes, both survival-related and with kME ≥0.8 and GeneSignificance >0.2 were regarded as hub genes in this study (22). The coexpression of hub genes was plotted by Cytoscape 3.6.0.

### Transcriptome deconvolution of the gene signatures

The abundance of infiltrating immune cell populations was estimated by deconvolution methods integrated in the R package “immunedeconv.” Other immune- or tumor-associated signatures in each sample were quantified by ssGSEA with the R package “GSVA.”

### Risk score model

We used univariate Cox regression, LASSO, and stepwise regression successively to screen out candidate mRNAs for construction. In the univariate Cox proportional risk regression analysis, mRNAs with p <0.05 was associated with survival. The criteria for LASSO regression remained in the model more than 900 times out of all 1,000 repetitions. Then step wise were used. The risk scoring model was constructed based on Cox coefficients and mRNAs’ expression. Risk score ∑I = 1 = (Coefi × Expri). The Expri represented the expression levels of mRNAs in the gene risk model, K–M survival analyses and ROC curves were performed to evaluate the predictive accuracy of models.

### Gene signature activity scores on cells

Specific gene sets’ activity scores for each cell type were calculated by AUCell (23). The gene set is the survival-related gene set of modules discussed in the WGCNA section. The scores were plotted as a heatmap and a violinplot.

### Cell–cell communication

CellPhoneDB (https://www.cellphonedb.org/) was used to infer the ligand–receptor crosstalk between single cells (24), which interpreted interactions in single cells based on known protein–protein interaction annotations. The number of ligand–receptors at intercellular junctions was calculated. As for the differential cell crosstalk analysis in each group, it was computed separately. The differential crosstalk between cells was visualized. Ligand activity was predicted by NicheNet (V1.1.0) (25).

### Statistical analysis

Hierarchical clustering analysis was performed on the R “hclust” function using the “ward.D” method to identify the number of subtypes in TCGA-GBM based on the pattern of signature scores. Univariate and multivariate Cox proportional hazards regression models were used to assess the association between the risk model and overall survival with and without clinical variables. The hazard ratio (HR) and 95% confidence interval (CI) were calculated. Wilcoxon rank sum, or Student tests, were used to compare two groups. For comparisons of more than two groups, one-way ANOVA tests and Kruskal–Wallis tests were utilized as parametric and nonparametric methods, respectively. The Kaplan–Meier method and log-rank test were conducted to compare survival differences between two groups. All statistical analysis was performed using R (version 4.0).

## Results

### Cytokine–cytokine receptor interaction tops the GBM risk factors

**Figure 1:**
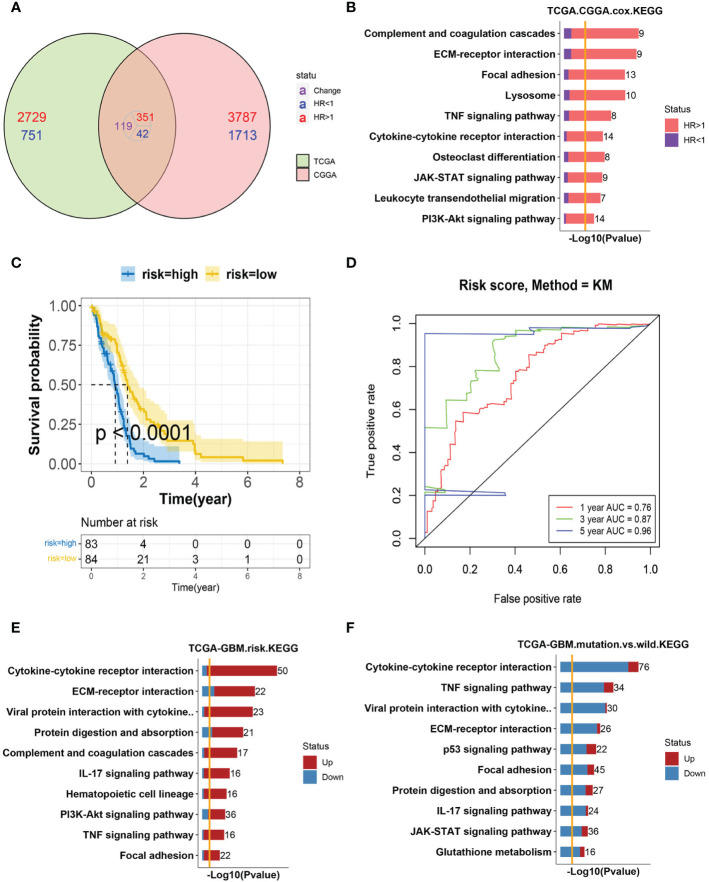
Analysis of GBM progression-related pathways **(A)** The Venn diagram of survival-related genes in the TCGA–GBM and CGGA cohorts. A total of 86 genes were found to coexist with diversity, of which 81 genes were associated with a poor prognosis. **(B)** Bar-plot of KEGG enrichment analysis of 86 survival-related genes with x-axis as −log10 transformed P-value. Bars were colored by the ratio of poor and good prognosis-related genes. **(C)** The Kaplan–Meier curves comparing patients with a low- or high-risk score in the TCGA–GBM cohort. Patients were divided into two groups according to the median value of their risk scores. Higher risk scores were correlated with a poorer prognosis. **(D)** ROC curve for the risk model in the TCGA–GBM cohort. **(E)** Bar-plot of KEGG enrichment analysis of DEGs between high- and low-risk groups. Bars were colored by the ratio of up and downregulated genes. Upregulated genes were those with elevated expression in the high-risk group. **(F)** Bar-plot of KEGG enrichment analysis of DEGs between mutant and wild-type patients. Bars were colored by the ratio of up and downregulated genes.

A univariate Cox hazard regression analysis was performed for all expressed genes in the TCGA–GBM cohort. We found 1264 genes as survival related in the TCGA–GBM cohort genes and 2,681 genes in the CGGA cohort (<0.01). There were 86 genes associated with survival in the two datasets (**Figure 1A**). The enriched KEGG pathways of these 86 genes were shown (**Figure 1B**). The relationship between these enriched pathways and GBM has been reported in several publications (11, 26, 27).

To further verify the predictive role of these genes in GBM progression, a risk model was constructed. Eight genes met the requirement through the least absolute shrinkage and selector operation (LASSO) regression. After stepwise regression, a model based on the expression of eight genes in the TCGA-GBM cohort was established. Patients in the high-risk group had a worse prognosis than those in the low-risk group (lop-rank test, p <0.001, **Figure 1C**). The area under the curve (AUC) was higher than 0.75 according to the ROC curves of the 1-, 3-, and 5-year OS predictions (**Figure 1D**), which means that the risk model has high predictive power. The risk model was validated in the CGGA cohort (**Figures S1A, B**). Then, the differentially expressed genes (DEGs) between the high- and low-risk groups were calculated in the TCGA–GBM cohort. These DEGs were also enriched in the pathways discussed above, among which cytokine–cytokine receptor interaction was the top one (**Figure 1E**).

The isocitrate dehydrogenase (IDH1) gene represents a recurrent mutation in GBM patients, which was associated with good prognostic outcomes compared to wild-type counterparts (TCGA-GBM cohort, log-rank p <0.0001, **Figure S1C**) (12). The enriched, upregulated pathways in the above high-risk patients were downregulated in IDH1 mutation samples, which further validated their pro-tumor characteristics in GBM (**Figure 1F**). Interestingly, cytokine–cytokine receptor interaction, again, was at the top of enriched pathways between IDH1 mutation and wild-type patients. As reported, within the heterogeneous GBM microenvironment, tumor cells, normal brain cells, immune cells, and stem cells interact with each other through the complex cytokine network (12). Therefore, we included these cell types into consideration next to complicatedly delineate the microenvironment of GBM.

### Heterogeneous TME components were associated with tumor-promoting pathways

The tumor microenvironment (TME) is composed of resident fibroblasts, endothelial cells, pericytes, leukocytes, and the extracellular matrix (9). To classify TMEs using a transcriptomic-based analytical platform, gene expression signatures (GES) representing the major functional components and immune, stromal, and other cellular populations of the tumor were constructed (**Figure S2A**). We selected five tumor-promoting pathways from the above upregulated pathways in high-risk patients according to biological background knowledge. Then we analyzed their correlation with other TME signatures, such as MDSC and monocytes. The five tumor-promoting pathways were significantly positively correlated with other pro-tumor or angiogenesis-related signatures and negatively correlated with anti-tumor-related signatures (**Figure S2B**). Then, we examined their characteristics in GBM progression by univariate Cox regression analysis on these TME-related characteristics, and we found most signatures were in high HR (**Figure S2C**). In summary, we comprehensively analyzed TME gene signatures of GBM and found heterogeneous TME components were associated with tumor-promoting pathways.

### Identification of the immunosuppressive subtype of GBM through GES classifier

**Figure 2:**
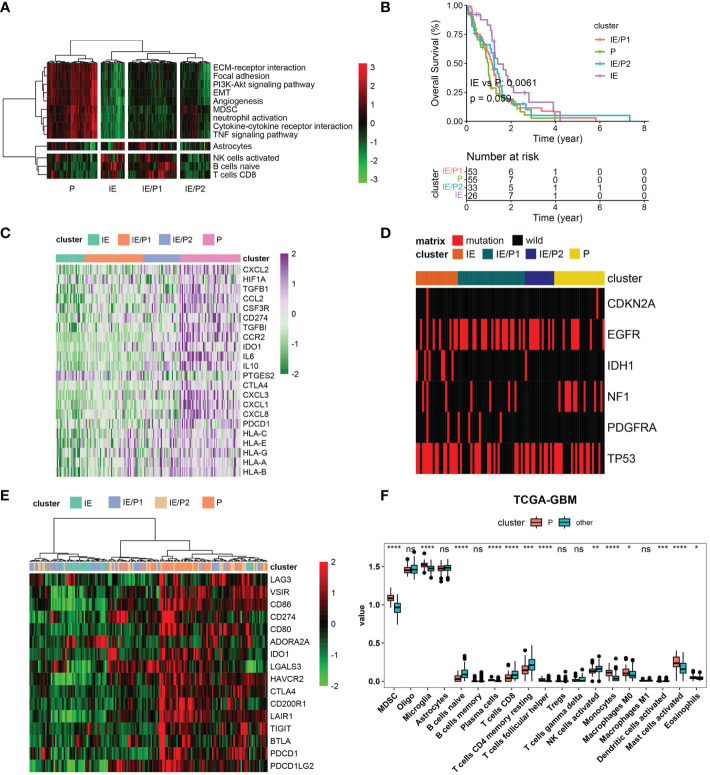
Immunosuppressive subtype identification in the TCGA–GBM cohort. **(A)** A heatmap of row-scaled gene signature scores from the cell deconvolution algorithm, with the color ranging from green to red, represents the activity score from low to high. The samples in this column were grouped into four TME subtypes. **(B)** Overall survival of patients stratified by TME subtype classification. The log-rank p-value between subtypes IE and P was 0.0061, and the annova log-rank p-value for four subtypes was 0.059. **(C)** The expression profile of immune suppression-related genes across four TME subtypes, with the color ranging from green to purple, represents the expression value from low to high. **(D)** Mutation frequency of five high-frequency mutant genes across four TME subtypes. Samples were shown in the column. Samples with mutations were color red. **(E)** The expression profile of inhibitory immune checkpoints across four TME subtypes. **(F)** Differential immune cell infiltration level across immunosuppressive subtypes and others. Statistical significance between groups was tested by Wilcox. *P < 0.05, **P < 0.01, ***P < 0.001, ****P < 0.0001, ns > = 0.05.

According to the expression activity of the selected GES in the TCGA–GBM dataset, patients were classified into four subtypes by the hierarchical clustering method. Based on the infiltrating situation of tumor killing cells and tumor progression characteristics, these subtypes were defined as tumor progression (P), immune infiltrating (IE), and expressing both simultaneously (P/IE) (**Figure 2A**). It was evident that the P subtype had higher tumor progression signatures and lower lymphocyte infiltration. These patients had the worst survival (**Figure 2B**, log-rank p = 0.0061). Then, we evaluated the differences between IE and P subtypes from several perspectives, such as immunosuppression, ICB, high-frequency mutation distribution, and cell infiltration. The expressions of immunosuppressive factors were plotted as a heat map (28), and it could be seen that subtype P represented higher expression of these genes (**Figure 2C**). For gene mutations, we plotted the distribution of five high-frequency mutations across the four subtypes (**Figure 2D**). IDH1 mutations were all of the IE type, which was consistent with their better outcomes. Tumor cells usually upregulate ICB gene expression to evade the immune system. We evaluated the expression of inhibitory ICBs in P-type cells (**Figure 2E**). In terms of cell infiltration, the P subtype showed high levels of myeloid cell infiltration and other subtypes showed high levels of lymphocyte infiltration (**Figure 2F**).

We validated these findings with the CGGA dataset. Four types were also found (**Figure S3A**). The log-rank p-value between IE and P subtypes was 0.00051 (**Figure S3B**). The P subtype also showed high expression of immunosuppressive factors and inhibitory ICBs (**Figures S3C, E**). Myeloid cells were infiltrated in subtype P, and lymphocytes were infiltrated in subtype IE (**Figure S3F**). Inconsistent with TCGA-GBM, IDH mutations were not predominantly distributed in the IE type but also in the IE/P type (**Figure S3D**). In conclusion, we identified immunosuppressive and lymphocyte subtypes both in the TCGA–GBM and CGGA cohorts and found their opposite biological characteristics.

### GBM subtypes represented heterogeneous functionalgene modules

**Figure 3:**
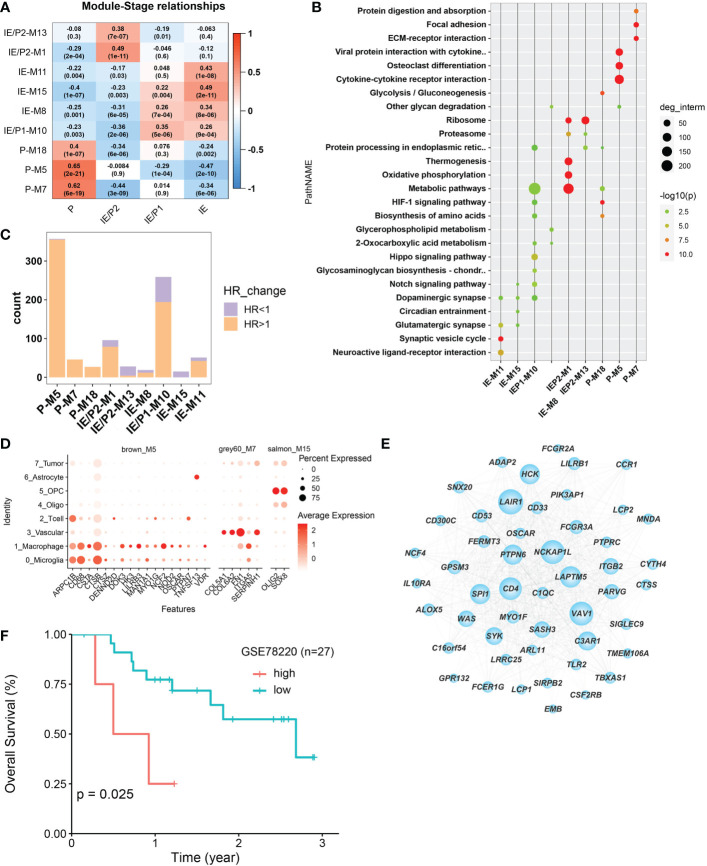
Co-expressed gene module detection by WGCNA. **(A)** Correlation between module eigengenes (1st principal component of modules) and TME subtypes in the TCGA–BGM cohort. The correlations were shown as a heatmap, gradually colored lower in blue and higher in red according to the Pearson correlation coefficient. The first line of the value in the heatmap represents the correlation coefficient, and the second line is the p-value from the correlation test. **(B)** Dotplot of the top enriched pathways of each module. Dots were colored gradually by −log10 (p-value), and the size of the dots gradually changed according to the number of genes contained, with the larger the value, the larger the dots. **(C)** Barplot shows the number of molecules that met the requirements of univariate Cox regression p-value <0.05 in each module. Bars were colored with an HR ratio >1 (orange) or <1 (purple). **(D)** The distribution of expression levels of each module’s survival-related hub genes across different cell types. The larger the size, the larger the percent of expression. **(E)** Co-expression network between the top 50 hub genes selected according to the kME score. The larger in node size the higher in node degree. The top 15 genes in degree were more important and were defined as Top15_hub. **(F)** Relationship between Top15_hub gene expression and prognosis of immunotherapy samples. The Kaplan–Meier curves comparing Top15_hub gene low and high expressed patients in an immunotherapy dataset (GSE78220).

The WGCNA algorithm was used to construct co-expressed gene modules (22). Twenty co-expressed modules were identified using the “cutreeHybrid” function (**Figure S4**). To find subtype-specific modules, we calculated the correlation between module genes and subtypes (**Figure 3A**). Genes in modules 7, 5, and 18 were highly expressed in the P subtype, while modules 1 and 13 were in the IE/P2 subtype, module 10 in the IE/P1 subtype, and modules 15, 8, and 11 were in the IE subtype. Functional enrichment analysis was performed on these subtype-specific modules, and the top 5 pathways with p-values ranking from small to large in each module were plotted (**Figure 3B**). M5 was enriched with genes participating in inflammatory responses, including cytokine interactions, chemokine signaling, and Th17 cell differentiation (**Figure S4C**). M7 was enriched with genes related to angiogenesis, including focal adhesion and PI3K/Akt signaling (**Figure S4D**). M18 was enriched with genes involved in the cellular response to hypoxia and carbon metabolism, including the HIF-1 signaling pathway and glycolysis/gluconeogenesis (**Figure S4E**). This suggested that these three different functionally related genes were involved in the formation of an immunosuppressive microenvironment. Both the IE/P2 and IE/P1 subtypes were related to metabolism. The IE subtype was mainly enriched in synapse and singling transduction-related pathways (**Figure S4F**). This indicated that the activity of the nervous system in the IE subtype was high.

The relationship between gene expression and patient survival in each module was analyzed by univariate Cox regression analysis. Genes with P <0.05 and HR >1 were considered pro-tumor-related genes, and genes with HR <1 were considered anti-tumor-related genes. The proportion of pro-tumor genes greater than 0.5 was considered a poor prognosis-related module. Similarly, the proportion of anti-tumor-related genes greater than 0.5 was considered to be prognosis-related. Finally, nine subtype-specific modules were divided into seven poor prognosis and two good prognosis-related modules (**Figure 3C**), and the survival-related genes of each subtype-specific module were abbreviated as ssMSGs (**Supplementary Table 2**, hubgene.survival.related.xlsx). A total of 24 hub genes (**Supplementary Table 3**, module cox logtpm.sel.xlsx) were obtained, which were mainly located in M5 and M7 (P subtype). These genes were mainly located in M5 and M7 (P subtype). Through co-expression analysis of top hub genes in different gene modules, we identified the top 15 hub genes in M5, which represented the top connections with each other (**Figure 3E**). The top 15 hub genes included LAPTM5, NCKAP1L, PTPN5, SYX, and SIGLEC9, which is consistent with the top risk pathways we concluded above. Further, the signatures of the top 15 hub genes signature were associated with poorer outcomes in immunotherapy cohorts, which is also consistent with the tumor-promoting function of M5 (**Figure 3F**).

Notably, compared with single-cell datasets, we confirmed that hub genes in M5, M7, and M15 were also marker genes for specific cell types. CSTs (CSTA, CSTB, and CSTZ), CD68, and NOD2 in M5 were markers of macrophages; COL6A2 and ITGA5 in M7 were related to vascular cells; and Oligo2 in M15 was a marker of oligodendrocytes (**Figure 3D**). This result indicated that specific cell types should represent different functional modules during GBM progression.Therefore, we turned to single-cell datasets in the next part to delineate GBM TME at the single-cell scale.

### Macrophages and microglia manipulate tumor-promoting gene modules of GBM

Next, we analyzed the expression activity of ssMSGs from nine subtype-specific modules in two published GBM single-cell datasets (GSE117891, GSE84465, and GSE163120) (18–20). GSE117891 and GSE84465 sequenced 10 patients’ single cells from both the tumor core and the peritumoral brain, including tumor cells, vessels, microglia, neurons, and glia. GSE163120 only detected immune cells; myeloid cells accounted for the majority. TAMs, blood vessels, and tumor cells were in the tumor core, while neurons and glial cells were mainly located in peripheral tissues. More immune cells were detected in recurrent samples (**Figure S5B**).

**Figure 4:**
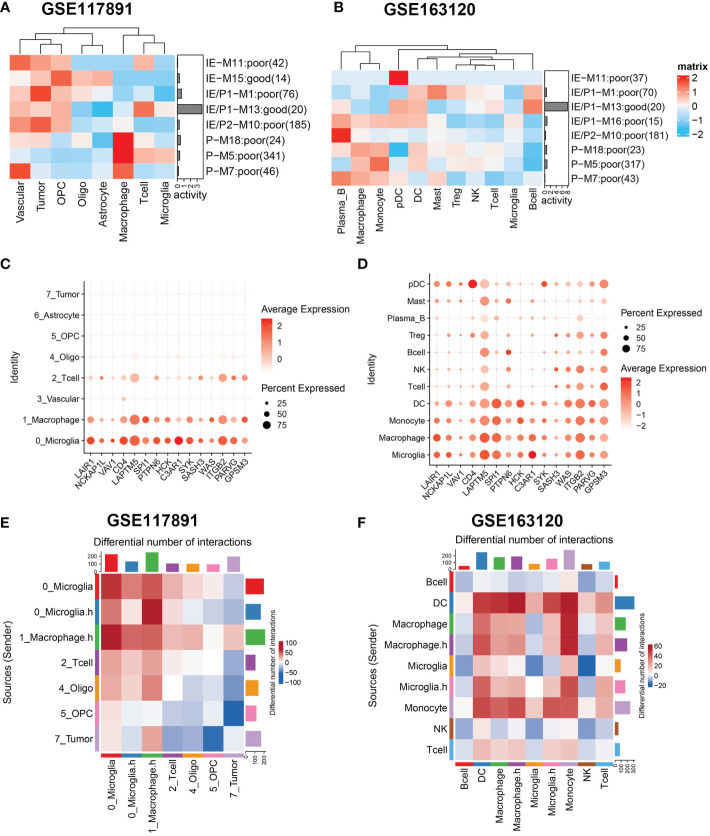
Gene sets expression activity and cell-cell interaction in sing-cell RNA-Seq datasets. **(A, B)** Row scaled gene expression activity of ssMSG across cell types in the GSE117891 and GSE163120 datasets, with the color from blue to red representing the activity score from low to high. Cells were clustered by the activity of these gene sets. **(C, D)** The expression level of Top15_hub genes across different cell types in two datasets. The larger the size, the larger the percent of expression. The darker the color, the higher the expression. **(E)** The differential cell–cell interaction weight between the tumor core and peripheral region of GSE117891.Upregulated interactions in tumor core were colored in red, down-regulated in blue. **(F)** The differential cell–cell interaction weight between recurrent and newly diagnosed samples of GSE163120. Upregulated interactions in recurrent samples were colored in red.

The “AUCcell” method was used to calculate the expression activity of ssMSGs in single cells (**Figures 4A, B**). As for the P subtype-related genes, M5 was highly expressed in myeloid cells, including TAMs, microglia, monocytes, and DCs; M7 was mainly expressed in blood vessels; and M18 in blood vessels, myeloid cells, and tumor cells. The IE subtype-related genes were in OPCs and neurons. The expression distribution of 24 hub genes across cells was shown (**Figures 4C, D**, **Figures S5C, D**). These genes were expressed in myeloid cells, blood vessels, and OPCs. Combined with these results, we concluded that macrophages manipulated M5, vascular-related cells contributed to M7, and OPC cells regulated M15.

### Cell–cell interaction

Considering the significant role of cell–cell interaction during GBM progression, we used cellphoneDB to figure out the interaction network of GBM (**Figures 4E, F**). We compared interaction strength in tumor samples with those of normal samples and found that macrophages exhibited high interaction with tumor cells among all cell types. This result was consistent with the characteristics of GBM tumor cells reported by others that they could interact with macrophages and induce their malignant transformation. Then we checked the interaction network among immune cells (**Figure 4F**). Interestingly, when we divided cells by expression of cytokine-related pathways, we found macrophages expressing higher cytokine pathways represented stronger interaction with DC and T cells, which may underline their pro-tumor mechanism. Similarly, we found microglia cells with higher cytokine pathway expression tend to interact with DC, macrophages, monocytes, and T cells. Specifically, Tregs showed stronger interaction with cytokine-high subtypes than their counterparts, which could reshape the immunosuppressive microenvironment (**Figure S5E**).

In summary, we identified specific cell types that manipulate different gene modules in GBM. We then focused on the interactions related to macrophages and microglia with other cell types in the microenvironment.

### Macrophage and microglia cells shape an immunosuppressive microenvironment through interaction with Tregs

**Figure 5:**
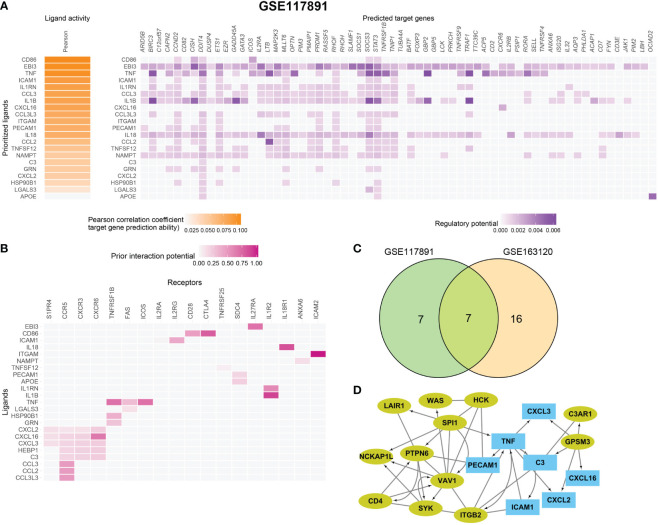
Macrophage and microglia cells shape an immunosuppressive microenvironment through interaction with Tregs in GSE163120. **(A)** A heatmap showing the predicted ligand activity by NicheNet on genes highly expressed in Treg. Pearson correlation indicates the ability of each ligand to predict the target genes, and better predictive ligands are thus ranked higher. **(B)** A dot heatmap showing the selected ligand-receptor pairs between macrophages or microglia and Treg cells. Benjamini–Hochberg adjusted permutation test. **(C)** The intersection between top ligands from both datasets, seven ligands in macrophage/microglia were detected. **(D)** protein–protein interaction between Top15_hub genes (colored in yellow green) and seven ligands (colored in sky blue) with both directed and undirected interaction. The directed interaction was shown in arrows. Some Top15_hub genes were involved in the regulation of ligands.

To further identify the key mediators of macrophage and microglia interaction in GBM patients, we use the R package “NicheNet” based on the expression and downstream targets of ligand–receptor pairs. Based on the above results, we chose Tregs for the following analysis (**Figures 5A, B**; **Figure S6**). We found that macrophages and microglia cells could directly contact Tregs through the adhesive ligand–receptor pairs ICAM1-IL2RG and ITGAM-ICAM2. In addition, macrophages and microglia cells enhanced the activation cytokine activity of tregs *via* the expression of EBI3, CD86, and TNF, inducing the expression of IL27RA, CD28, TNFRSF1B, FAS, ICOS, and the immune checkpoint CTLA4 on tregs. Additionally, macrophages and microglia cells enhanced the recruitment of tregs through CXCL16–CXCR6, CCL3–CCR5, CCL2–CCR5 pairs.

Then, we evaluated which ligands on macrophages or microglia cells could most likely regulate Tregs. We merged the GSE163120 and GSE117891 datasets and identified seven ligand genes (**Figure 5C**). The regulatory network between the top 15 hub genes and these ligands is shown in **Figure 5D**. SPI1 could be the upstream regulator of TNF, and GPSM3 may regulate the expression of a series of cytokines and chemokines such as C3, CXCL3, CXCL16, and CXCL2.

In conclusion, we find out how upstream regulators regulate ligand expression on macrophages and microglia cells, how ligands interact with their receptors on tregs, and how these interactions thus shape the immunosuppressive microenvironment of GBM.

## Discussion

The characteristics and mechanisms of the tumor microenvironment, especially the immunosuppressive microenvironment, in patients with GBM are still unclear. In addition to immunosuppressive microenvironment, in patients with GBM are still unclear. In addition to various immune cells’ infiltration, the tumor microenvironment also contains glial cells, vascular-related cells, fibroblasts, immunosuppressive factors, etc. The major signaling pathways also play a key role in the formation of GBM. On the research of tumor immune microenvironment, previous studies mainly focused on estimating the composition of immune cells or including some immune system-related signatures, while ignoring the role of non-immune factors. In addition, the cell type infiltration and signaling pathways involved were rarely the subjects of deeper discussions in previous studies. In this study, we first collected various functional signatures related to the GBM tumor microenvironment and divided GBM patients into four groups according to the expression profiles of these signatures. The immunosuppressive subtypes were successfully defined and which had elevated expression of immunosuppressive molecules such as IDO1, Il-6, etc. Then we conducted an in-depth study of the cellular composition and interaction of the immunosuppressive subtypes.

As reported, some major pathways played a key role in the tumor progression or influenced the formation of an immunosuppressive microenvironment in GBM (14–16, 29). For example, GBM cancer-related cytokine deregulation might be responsible for the failure of the immune system to recognize malignant tumor cells (11). The increase of pro-angiogenic growth factors, including VEGF, led to a high degree of tumor vascularization (30). In this study, five pathways that were significantly related with the GBM progression were found by analysis from three different perspectives. These three perspectives differed in methodology, but the results were indeed very consistent. This indicated that these pathways were very important in the progression of GBM. They were mainly involved in two directions: inflammatory response related, including TNF-α signaling and cytokine–cytokine interactions and angiogenesis related to ECM, focal adhesion and the PI3K/Akt signaling pathway. The activity of these five signaling pathways was positively correlated with the infiltration of myeloid suppressor cells (MDSCs), which were reported to participate in the immunosuppression of GBM (31). Therefore, we used the genes from these five pathways for further GBM subtyping.

Among the four GBM subtypes we found, these were immune-infiltrating (IE) and immunosuppressive (P). Statistical differences in survival were identified among the types of patients (long-rank p-value <0.01). As expected, the P subtype had high expression of ICB and immunosuppressive factors and no IDH mutation, while the IE subtype had high lymphocyte infiltration. Unexpectedly, in the IE subtype, we did not find the high expression of genes related to lymphocytes activation, but only synapse related genes were detected. It was reported that lymphocytes infiltrated in GBM were rarely activated, which might explain our findings. This suggested that immunotherapy targeting T cells in GBM might not be meaningful.

In addition, we were surprised to find that the three co-expressed gene modules associated with the P subtype differ greatly in enriched pathways according to the following WGCNA analysis. These three gene modules had the functions of inflammatory response (cytokine interaction), angiogenesis, hypoxia, and carbon metabolism, respectively. This indicated that three different functional genes worked together to influence the formation of the P subtype. By verifying the expression of ssMSGs in two publicly available single-cell datasets, we found that three modules corresponded to different types of cells (TAM, blood vessels, tumors). Therefore, we inferred that these types of cells worked together to form the immunosuppressive microenvironment. Also, we found that TAM and tumor had significant interactions in the tumor core through cell interaction analysis. More interestingly, we found novel hub genes from immunosuppressive modules could be the upstream regulators of a series of cytokines and chemokines such as C3, CXCL3, CXCL16, and CXCL2 in macrophages and microglia cell, which further interact with Treg and shape the immunosuppressive microenvironment of GBM.

In conclusion, we combined bulk- and single-cell RNA-seq data to profile the GBM tumor microenvironment using bioinformatics tools, and discovered important cells and pathways involved in the formation of the tumor immunosuppressive microenvironment (Graphic abstract). Future research needs to focus on inhibiting the interference signaling pathways in myeloid cells, especially TAM cells and the interaction between Tregs, which may be a beneficial therapeutic direction for GBM tumors.

## Data availability statement

The datasets presented in this study can be found in online repositories. The names of the repository/repositories and accession number(s) can be found in the article/**Supplementary Material**.

## Author contributions

LN and BL conceived and designed the study. LN, PS, and SZ performed the analysis flowchart and collected the data. BQ, XC, and MX contributed to analyzing the data. LN wrote the manuscript. BL made manuscript revisions. All authors have read and approved the manuscript.

## Supplementary material

Supplementary Table 1 The detail clinic pathological characteristics for TCGA-GBM and CGGA cohort.

Supplementary Table 2 The survival related genes of each subtype specific module; which were mainly located in M5 and M7 (P subtype).

Supplementary Figure 1 Risk model in CGGA-cohort **(A)** The Kaplan–Meier curves comparing patients with low or high risk score in CGGA cohort. Patients were divided into two groups according to the median value of risk scores. Higher risk score were correlated to poorer prognosis. **(B)** ROC curve for risk-model in CGGA cohort. **(C)** The Kaplan–Meier curves comparing IDH1 mutation and wild-type patients in TCGA-GBM cohort.

Supplementary Figure 2 Generation of the Ges utilized for transcriptomic-based TME classification **(A)** The 13 Gges included in each functional group. **(B)** Correlation analysis between signatures in TCGA-GBM cohort. Positive correlation coefficient was shown in orange and negative correlation coefficient was shown in blue, darker color indicates bigger value. **(C)** Result of univariate Cox regression analysis in TCGA-GBM cohort. HR and p values were displayed.

Supplementary Figure 3 Immunosuppressive subtype validation in CGGA cohort **(A)** Heatmap of row scaled gene signature scores from cell deconvolution algorithm with the color from green to red represents the activity score from low to high. Samples in column were grouped into four TME subtypes. **(B)** Overall survival of patients stratified by TME subtype classification. The log-rank p-value between subtype IE and P was 0.0051 and the annova log-rank p-value for four subtypes was 0.0001. **(C)** The expression profile of immune suppression related genes checkpoints across TCGA-GBM four TME subtypes with the color from green to purple represents the expression value from low to high. **(D)** Mutation frequency of five high frequency mutant gene across four TME subtypes. Samples were shown in column. Samples with mutation were colored in red. **(E)** The expression profile of inhibitory immune checkpoints across four TME subtypes. **(F)** Differential immune cell infiltration level across Immunosuppressive subtype and other’s. Statistical significance between groups was tested by Wilcox.

Supplementary Figure 4 WGCNA construction **(A)** Determine soft-thresholding power in WGCNA. The scale-free fit index for various soft-thresholding powers (β) (Left). The mean connectivity for various softthresholding powers (Right). **(B)** WGCNA cluster dendrogram on TCGA-GBM patients, genes were grouped into several distinct modules. **(C, D, E, F)** Top10 enriched pathways of 4 selected modules, M5 **(C)**, M7 **(D)**, M18 **(E)**, M15 **(F)**.

Supplementary Figure 5 Gene expression validation in 2 sing-cell RNA-Seq datasets **(A)** The ratio of cell types between tumor core (T) and peripheral region (N) of GSE117891. **(B)** The ratio of cell types between recurrent (R) and newly diagnosed (ND) samples of GSE163120. **(C, D)** Violin plots of selected pathways’ expression activity across cell-types with y-axis as expression activity in two datasets. (E) The interaction weight between Treg and other immune cells in recurrent samples of GSE163120. The thick in line the bigger in weight.

Supplementary Figure 6 Cell interaction between macrophage/microglia and T cell in GSE117891 Heatmap showing the predicted ligand activity by NicheNet on genes highly expressed in Treg. Pearson correlation indicates the ability of each ligand to predict the target genes, and better predictive ligands are thus ranked higher.
