# Supplementary material for: Transcriptome and single-cell analysis reveal the contribution of immunosuppressive microenvironment for promoting glioblastoma progression
Source: Front Immunol. 2023 Jan 5;13:1051701. doi: 10.3389/fimmu.2022.1051701 (PMC9851159; doi:10.3389/fimmu.2022.1051701)
Supplement: Supplementary Table 1 — The detail clinic pathological characteristics for TCGA-GBM and CGGA cohort. [file Table_1.docx]

TableS1:

TCGA cohort

| **term** | **count** | **percent** |
| --- | --- | --- |
| Gender |  | |
| FEMALE | 59 | 35.33% |
| MALE | 108 | 64.67% |
| vital_status |  | |
| Alive | 52 | 31.14% |
| Dead | 114 | 68.26% |
| Unknown | 1 | 0.60% |
| cancer_status |  | |
| TUMOR FREE | 13 | 7.78% |
| Unknown | 18 | 10.78% |
| WITH TUMOR | 136 | 81.44% |
| Age |  | |
| old | 88 | 52.69% |
| young | 79 | 47.31% |

CGGA cohort

| Term | count | percent |
| --- | --- | --- |
| Histology |  | |
| GBM | 198 | 55.62% |
| rGBM | 128 | 35.96% |
| sGBM | 30 | 8.43% |
| Grade |  | |
| WHO IV | 356 | 100.00% |
| Gender |  | |
| Female | 144 | 40.45% |
| Male | 212 | 59.55% |
| Age |  | |
| old | 77 | 21.63% |
| young | 279 | 78.37% |
| Chemo_status |  | |
| treated | 277 | 77.81% |
| Unknown | 21 | 5.90% |
| untreated | 58 | 16.29% |
| IDH_mutation_status |  | |
| Mutant | 85 | 23.88% |
| Unknown | 10 | 2.81% |
| Wildtype | 261 | 73.31% |
