# Supplementary figures and images for: Transcriptome and single-cell analysis reveal the contribution of immunosuppressive microenvironment for promoting glioblastoma progression
Source: Front Immunol. 2023 Jan 5;13:1051701. doi: 10.3389/fimmu.2022.1051701 (PMC9851159; doi:10.3389/fimmu.2022.1051701)

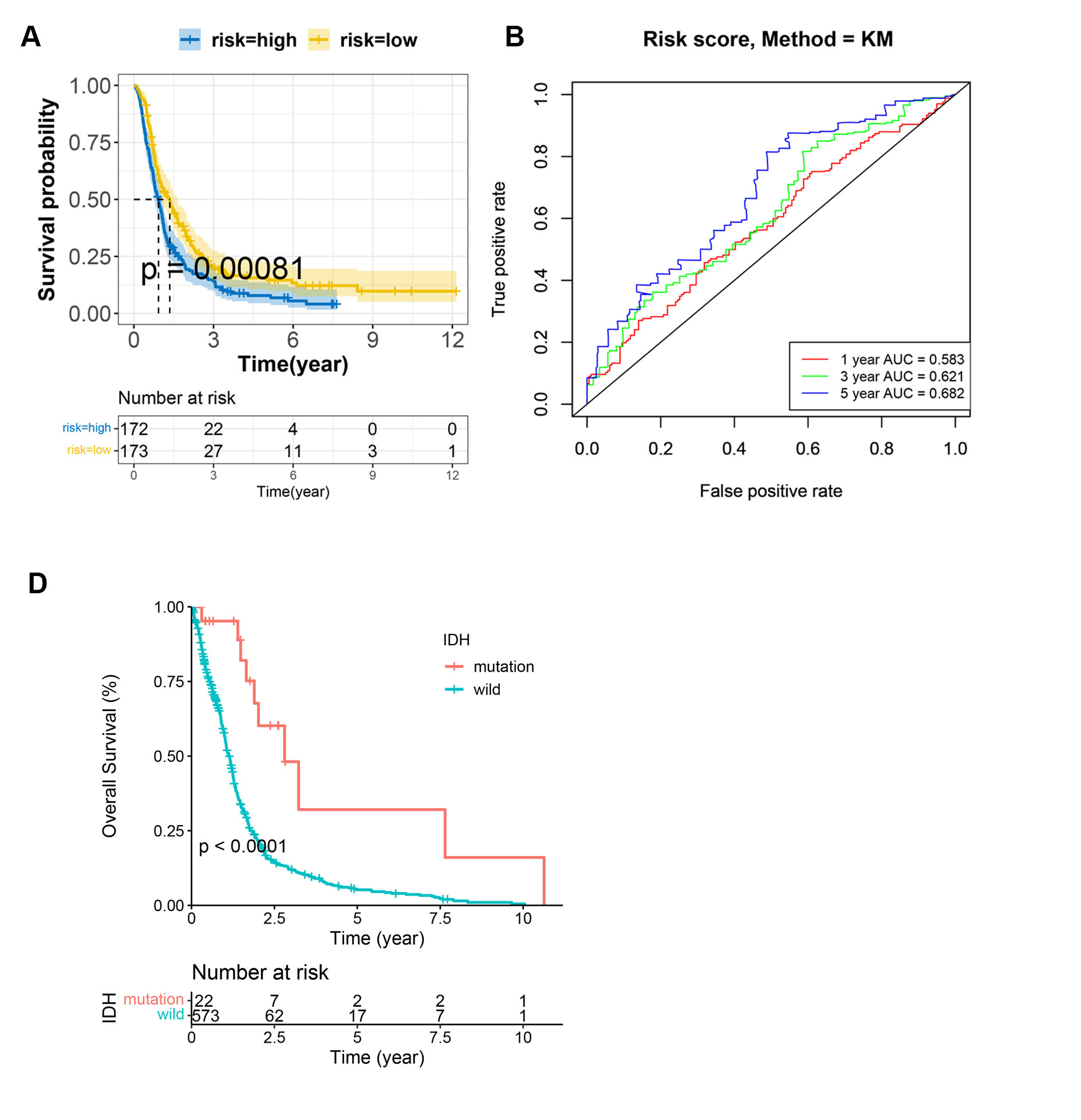

Supplement: Supplementary Figure 1 — Risk model in CGGA-cohort (A) The Kaplan–Meier curves comparing patients with low or high risk score in CGGA cohort. Patients were divided into two groups according to the median value of risk scores. Higher risk score were correlated to poorer prognosis. (B) ROC curve for risk-model in CGGA cohort. (C) The Kaplan–Meier curves comparing IDH1 mutation and wild-type patients in TCGA-GBM cohort. [file Image_1.jpeg]

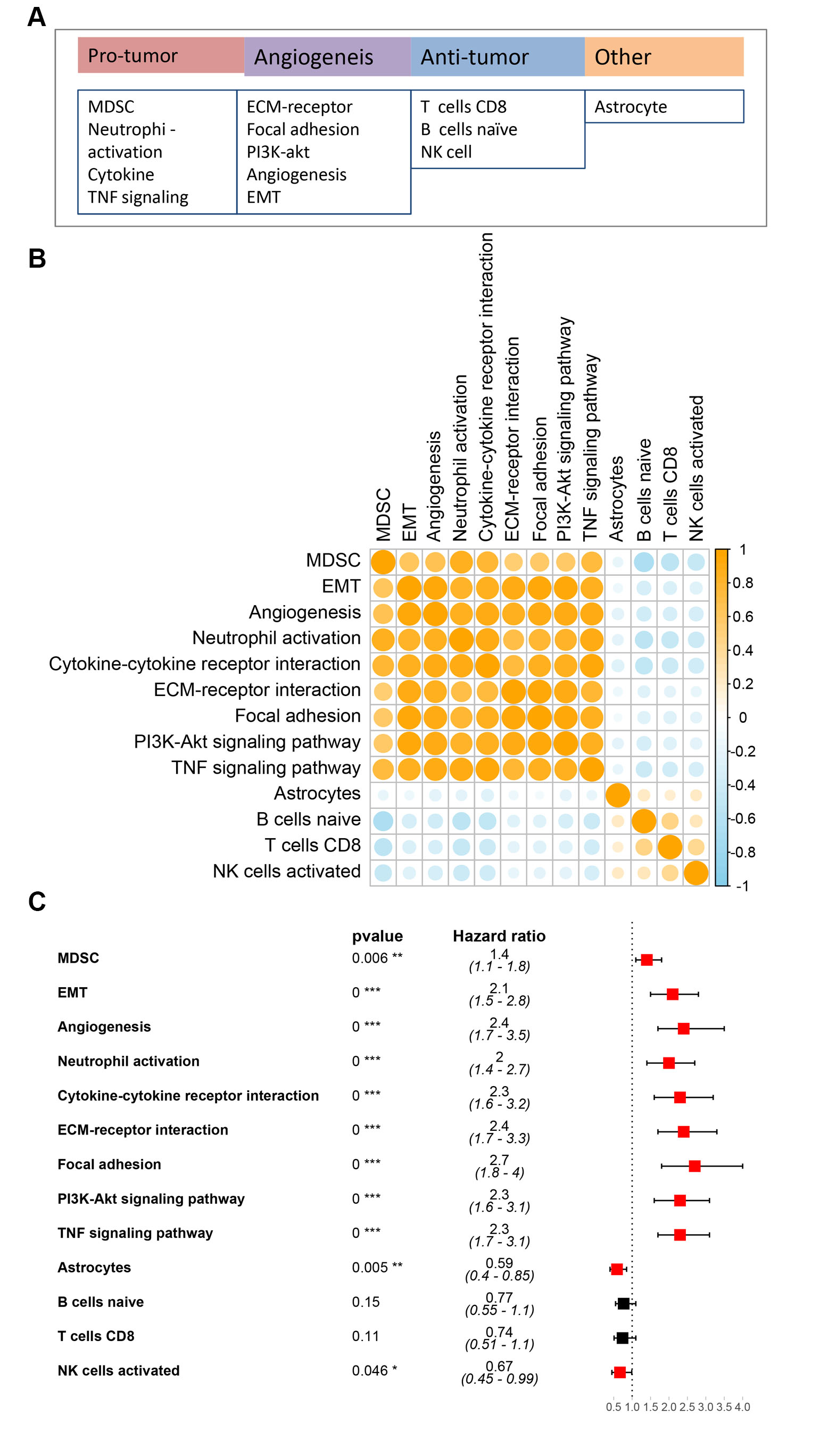

Supplement: Supplementary Figure 2 — Generation of the Ges utilized for transcriptomic-based TME classification (A) The 13 Gges included in each functional group. (B) Correlation analysis between signatures in TCGA-GBM cohort. Positive correlation coefficient was shown in orange and negative correlation coefficient was shown in blue, darker color indicates bigger value. (C) Result of univariate Cox regression analysis in TCGA-GBM cohort. HR and p values were displayed. [file Image_2.jpeg]

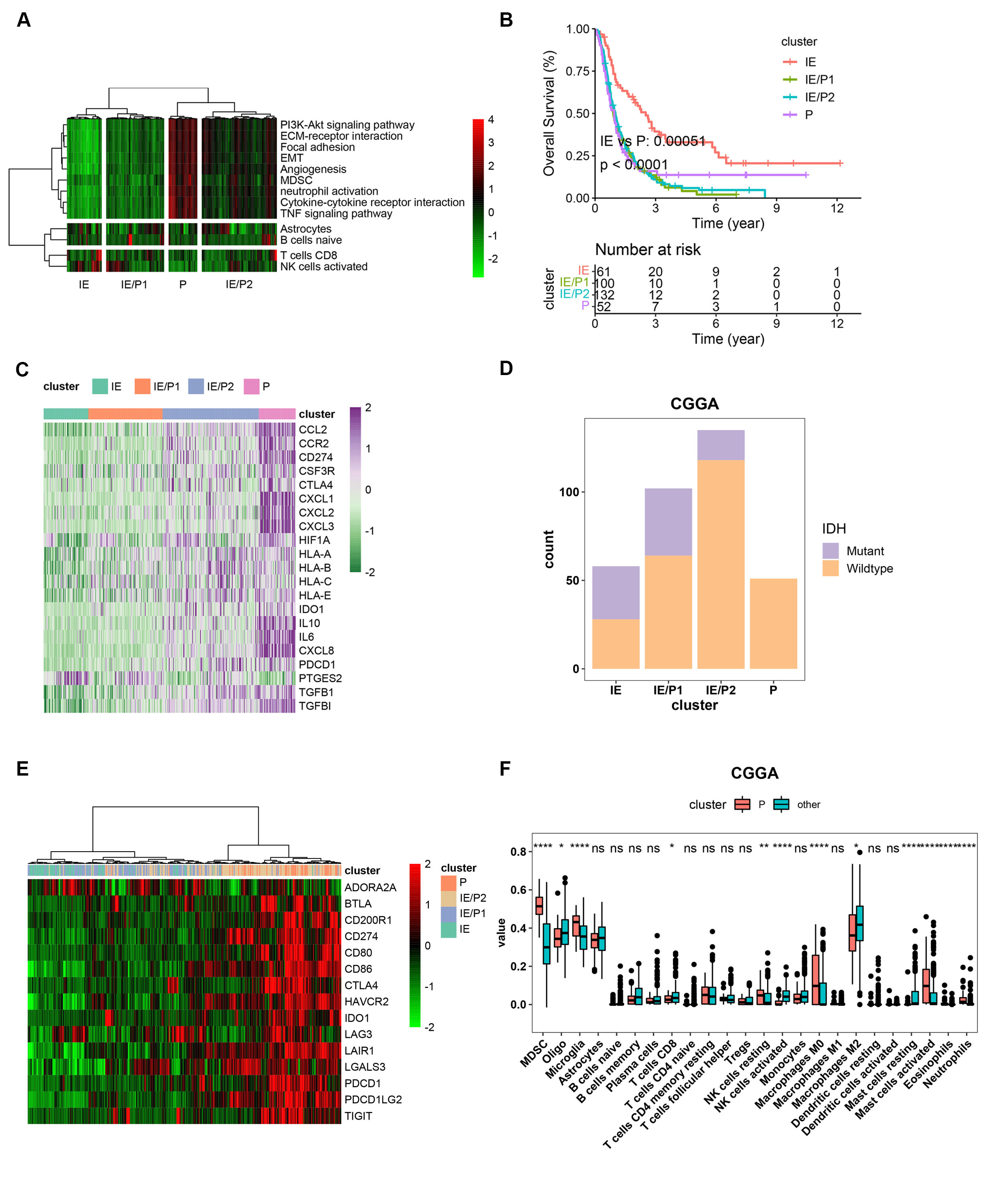

Supplement: Supplementary Figure 3 — Immunosuppressive subtype validation in CGGA cohort (A) Heatmap of row scaled gene signature scores from cell deconvolution algorithm with the color from green to red represents the activity score from low to high. Samples in column were grouped into four TME subtypes. (B) Overall survival of patients stratified by TME subtype classification. The log-rank p-value between subtype IE and P was 0.0051 and the annova log-rank p-value for four subtypes was 0.0001. (C) The expression profile of immune suppression related genes checkpoints across TCGA-GBM four TME subtypes with the color from green to purple represents the expression value from low to high. (D) Mutation frequency of five high frequency mutant gene across four TME subtypes. Samples were shown in column. Samples with mutation were colored in red. (E) The expression profile of inhibitory immune checkpoints across four TME subtypes. (F) Differential immune cell infiltration level across Immunosuppressive subtype and other’s. Statistical significance between groups was tested by Wilcox. [file Image_3.jpeg]

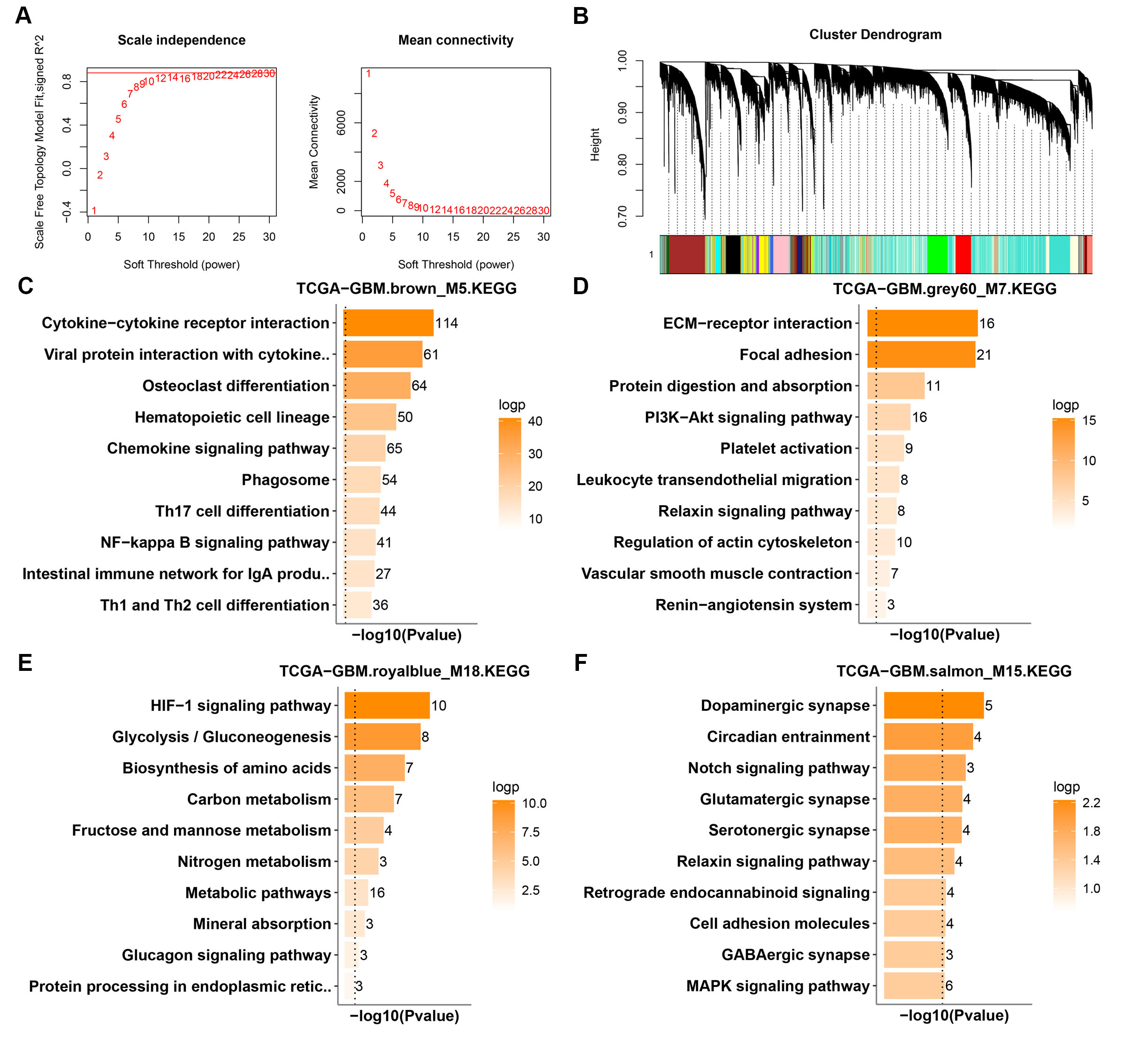

Supplement: Supplementary Figure 4 — WGCNA construction (A) Determine soft-thresholding power in WGCNA. The scale-free fit index for various soft-thresholding powers (β) (Left). The mean connectivity for various softthresholding powers (Right). (B) WGCNA cluster dendrogram on TCGA-GBM patients, genes were grouped into several distinct modules. (C, D, E, F) Top10 enriched pathways of 4 selected modules, M5 (C), M7 (D), M18 (E), M15 (F). [file Image_4.jpeg]

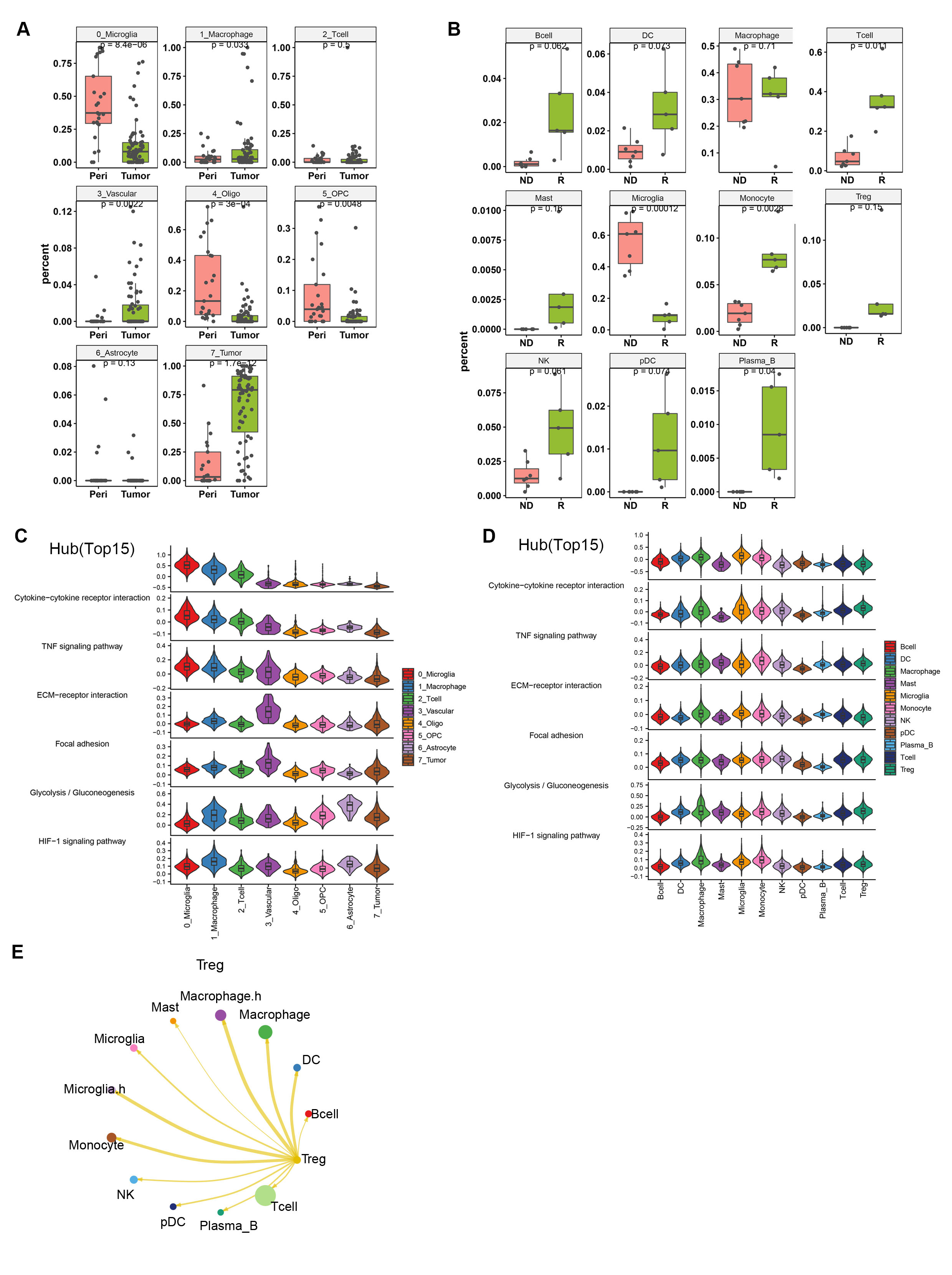

Supplement: Supplementary Figure 5 — Gene expression validation in 2 sing-cell RNA-Seq datasets (A) The ratio of cell types between tumor core (T) and peripheral region (N) of GSE117891. (B) The ratio of cell types between recurrent (R) and newly diagnosed (ND) samples of GSE163120. (C, D) Violin plots of selected pathways’ expression activity across cell-types with y-axis as expression activity in two datasets. (E) The interaction weight between Treg and other immune cells in recurrent samples of GSE163120. The thick in line the bigger in weight. [file Image_5.jpeg]

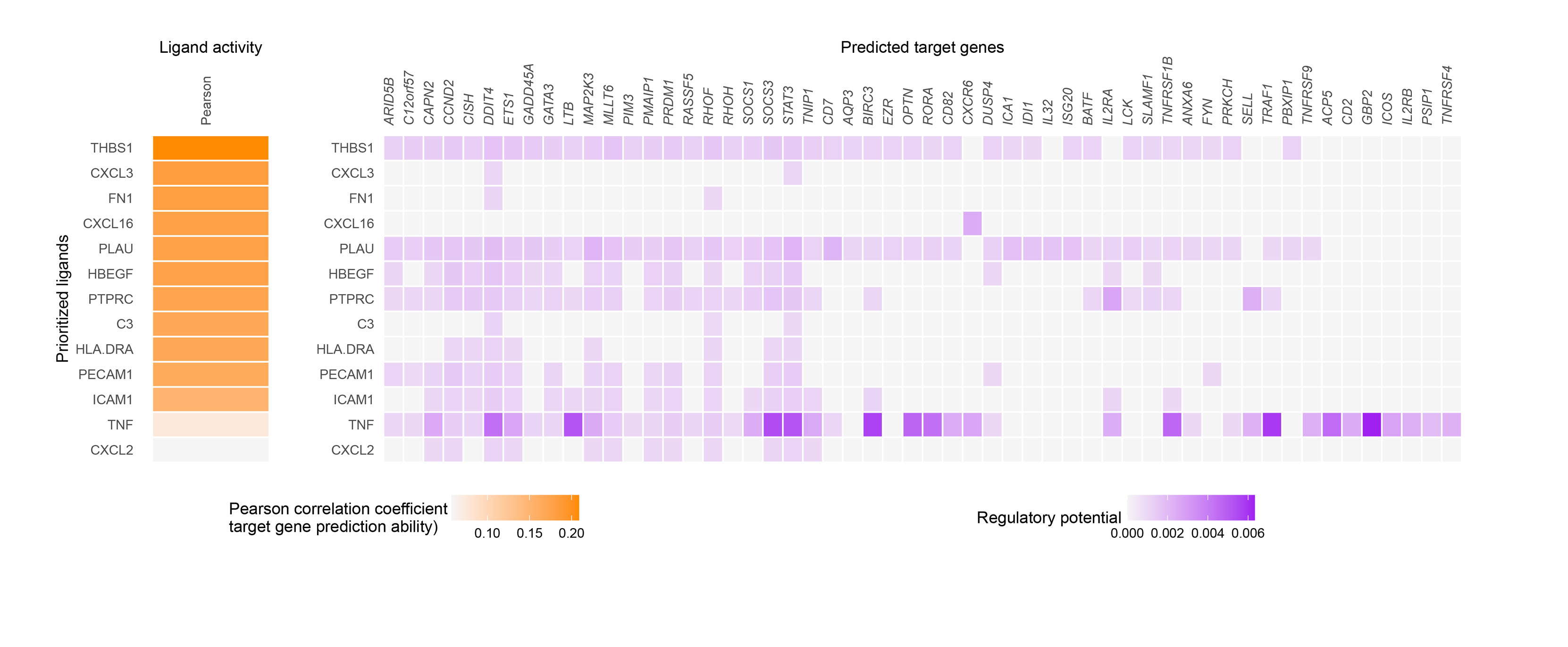

Supplement: Supplementary Figure 6 — Cell interaction between macrophage/microglia and T cell in GSE117891 Heatmap showing the predicted ligand activity by NicheNet on genes highly expressed in Treg. Pearson correlation indicates the ability of each ligand to predict the target genes, and better predictive ligands are thus ranked higher. [file Image_6.jpeg]
